# Machine Learning Algorithms for Predicting Surgical Outcomes after Colorectal Surgery: A Systematic Review

**DOI:** 10.1007/s00268-022-06728-1

**Published:** 2022-09-15

**Authors:** Mustafa Bektaş, Jurriaan B. Tuynman, Jaime Costa Pereira, George L. Burchell, Donald L. van der Peet

**Affiliations:** 1 Department of Surgery, Amsterdam UMC Location Vrije Universiteit Amsterdam, De Boelelaan 1117, 1081 HV Amsterdam, The Netherlands; 2 Department of Computer Science, Vrije Universiteit Amsterdam, De Boelelaan 1105, 1081 HV Amsterdam, The Netherlands; 3 Medical Library, Amsterdam UMC Location Vrije Universiteit Amsterdam, De Boelelaan 1117, 1081 HV Amsterdam, The Netherlands

## Abstract

**Background:**

Machine learning (ML) has been introduced in various fields of healthcare. In colorectal surgery, the role of ML has yet to be reported. In this systematic review, an overview of machine learning models predicting surgical outcomes after colorectal surgery is provided.

**Methods:**

Databases PubMed, EMBASE, Cochrane, and Web of Science were searched for studies using machine learning models for patients undergoing colorectal surgery. To be eligible for inclusion, studies needed to apply machine learning models for patients undergoing colorectal surgery. Absence of machine learning or colorectal surgery or studies reporting on reviews, children, study abstracts were excluded. The Probast risk of bias tool was used to evaluate the methodological quality of machine learning models.

**Results:**

A total of 1821 studies were analysed, resulting in the inclusion of 31 articles. A vast proportion of ML algorithms have been used to predict the course of disease and response to neoadjuvant chemoradiotherapy. Radiomics have been applied most frequently, along with predictive accuracies up to 91%. However, most studies included a retrospective study design without external validation or calibration.

**Conclusions:**

Machine learning models have shown promising potential in predicting surgical outcomes after colorectal surgery. However, large-scale data is warranted to bridge the gap between calibration and external validation. Clinical implementation is needed to demonstrate the contribution of ML within daily practice.

**Supplementary Information:**

The online version contains supplementary material available at 10.1007/s00268-022-06728-1.

## Introduction

Colorectal cancer is estimated to have approximately 2 million new cases and 1 million deaths per year [1]. Appendicitis cases appeared to be approximately 18 million in the last few years [2]. Performing colorectal surgical procedures come with several risks, such as postoperative bleeding, anastomotic leakage, or fistulas [3]. These complications could become a burden for surgeons because they lead to readmissions of patients and require revision surgery. Additionally, in patients with colorectal cancer, tumor recurrence or metastasis are commonly discovered, causing a decrease in survival for these patients [4]. Although chemotherapy has already demonstrated improvements in survivability for colorectal cancer patients, it is still difficult to predict which patients will completely respond to chemotherapy [5]. Therefore, risk stratification of patients with colorectal cancer remains challenging. Artificial Intelligence (AI) could support surgeons with this risk stratification by predicting postoperative complications, response to chemotherapy, and overall survival of colorectal cancer patients.

Recently, machine learning (ML), an essential branch of AI, has already been used for several complex tasks within healthcare. Examples of these tasks are the detection of tumors on radiologic images and prediction of biomarkers [6]. Due to its ability to train on large datasets and recognize patterns within data, machine learning algorithms are able to improve the accuracy of their prediction model [7]. Based on this capacity, machine learning models could be used to predict surgical outcomes prior to colorectal surgery [8]. By assessing several surgical outcomes with AI, surgeons could preoperatively decide the most efficient clinical pathway for patients undergoing colorectal surgery [9]. Currently, there are several machine learning algorithms available to make these predictions, an overview of algorithms is presented in Table 1.

**Table 1:** Terminology of AI subsets

Machine learning (ML)	Algorithms that are able to improve the prediction accuracies by training on large data [10]
Decision tree	A model that consists of nodes and branches, representing variables and related outcomes. Various combinations of outcomes give several predictions. The end model will be the smallest tree that fits the data best [11]
Gradient boosting (GBM)	Builds models that focus on inaccuracies of preceding models and improves these parts until the most accurate model is formed [12]
Random forest	Combines multiple decision trees to build the final accurate prediction model [13]
Support vector machine (SVM)	Finds the optimal border in the dataset to classify outcomes in two groups [14]
Artificial neural networks (ANNs)	Trains by using various processing layers to automatically find relevant features for the prediction. Additionally, weights of the extracted features are adjusted to form the most accurate model [15]
Convolutional neural networks (CNNs)	Similar to ANNs, except these models use filters instead of weight for extracted features [16]
Deep learning	Deep learning algorithms function similarly to neural networks, however, deep learning models have more layers or depth than neural networks [17]
Radiomics	Extracts quantitative features of clinical images to construct predictive or prognostic associations with the predicted medical outcomes [18]

*ML* machine learning, *SVM* support vector machine, *GBM* gradient boosting machine, *RF* random forest, *ANN* artificial neural networks, *CNN* convolutional neural networks

Although machine learning algorithms have shown major potential to improve surgical outcomes, the current status and quality of machine learning models within colorectal surgery have not been evaluated in recent literature. However, it is essential to bridge this gap in order to understand the extent of predicted surgical outcomes, generalizability, and validity of current machine learning algorithms applied in colorectal surgery. Therefore, this systematic review aims to provide a comprehensive overview of machine learning algorithms that have been used to predict any surgical outcome after general colorectal surgery. This review also evaluates the area under the curve and/or accuracy of included machine learning models.

## Materials and methods

Literature was retrieved and systematically reviewed in accordance with the Cochrane Handbook for Systematic Reviews of Interventions version 6.0 and PRISMA guidelines.

### Literature search strategy

A systematic search was performed in the databases: PubMed, Embase.com, Clarivate Analytics/Web of Science Core Collection and the Wiley/Cochrane Library. The timeframe within the databases was from inception to the 7th of July 2021 and conducted by G.L.B. and M.B. The search included keywords and free text terms for (synonyms of) 'machine learning' combined with (synonyms of) 'digestive system surgical procedures'. This search strategy was peer-reviewed by an information specialist (G.L.B.), using the PRESS checklist. A full overview of the search terms per database can be found in the supplementary information (see Appendix 1 as ESM). No limitations on date were applied in the search. Studies reporting on conference proceedings, book chapters, editorials, errata, letters, notes, surveys, or tombstones were excluded from the search.

### Eligibility criteria

Studies were only eligible if they specifically met the following criteria: (i) described machine learning methods, (ii) involved patients undergoing any type of colorectal surgery, (iii) reported predictive performance of the machine learning model, (iv) clinical study. Regression models could be seen as machine learning. Nonetheless, regression models have existent in healthcare for many years. As this review is addressing new machine learning models only, regression models are therefore excluded from this review. In addition, appendectomy procedures were considered as colorectal surgery. Studies were excluded if they (i) were not written in English, (ii) reported on reviews, editorials, letters, or study abstracts. No specific study design or setting was preferred in the inclusion criteria.

### Study selection

Two reviewers (M.B. & J.C.P.) independently performed the title and abstract screening in conformity with the inclusion and exclusion criteria. Eligible articles were read in full text, and duplicate studies were eliminated. The full-text screening of the retrieved articles was performed by the same two reviewers (M.B. & J.C.P.) to secure they comply with the inclusion criteria. Disagreements were resolved by discussions between two reviewers, resulting in consensus.

### Risk of bias assessment

The Probast risk of bias tool was independently applied to each study by two reviewers (M.B. & J.C.P.) to assess the methodological quality of included machine learning models [19]. This tool is able to evaluate the overall risk of bias based on four bias domains: participant selection, predictors, outcomes, and analysis.

### Data collection process

A table was formed for the extraction of all data. All data aspects were independently extracted and double-checked by two of the authors (M.B. & J.C.P.). Conflicts were resolved by consensus between the two authors. No additional processes were required for this data.

### Data items

An inventory of data items was formed according to the Cochrane guidance for data collection, and the CHARMS checklist [20]. The following information was extracted from each study: first author, publication year, country of research, number of patients, mean age, study design, surgical procedure, intervention, surgical outcome, internal validation method, external validation, predictive performance (discrimination, and calibration). For studies involving multiple machine learning models, predictive performance of each model was described separately.

### Data synthesis

A descriptive summary was used to represent the type of machine learning models, predicted surgical outcomes, risk of bias assessment, and model validation. To illustrate the predictive performance of machine learning models, results of machine learning studies were reported for each predicted outcome. To represent the discriminative ability, the range of mean accuracy (ACC) and area under the curve (AUC) was described for machine learning models of each predicted outcome. Additionally, the proportion of machine learning models that have applied calibration was described, along with the calibration method. A comparative meta-analysis of machine learning models was not possible, due to heterogeneity in study methodology, and the report on outcomes.

## Results

**Fig. 1:**
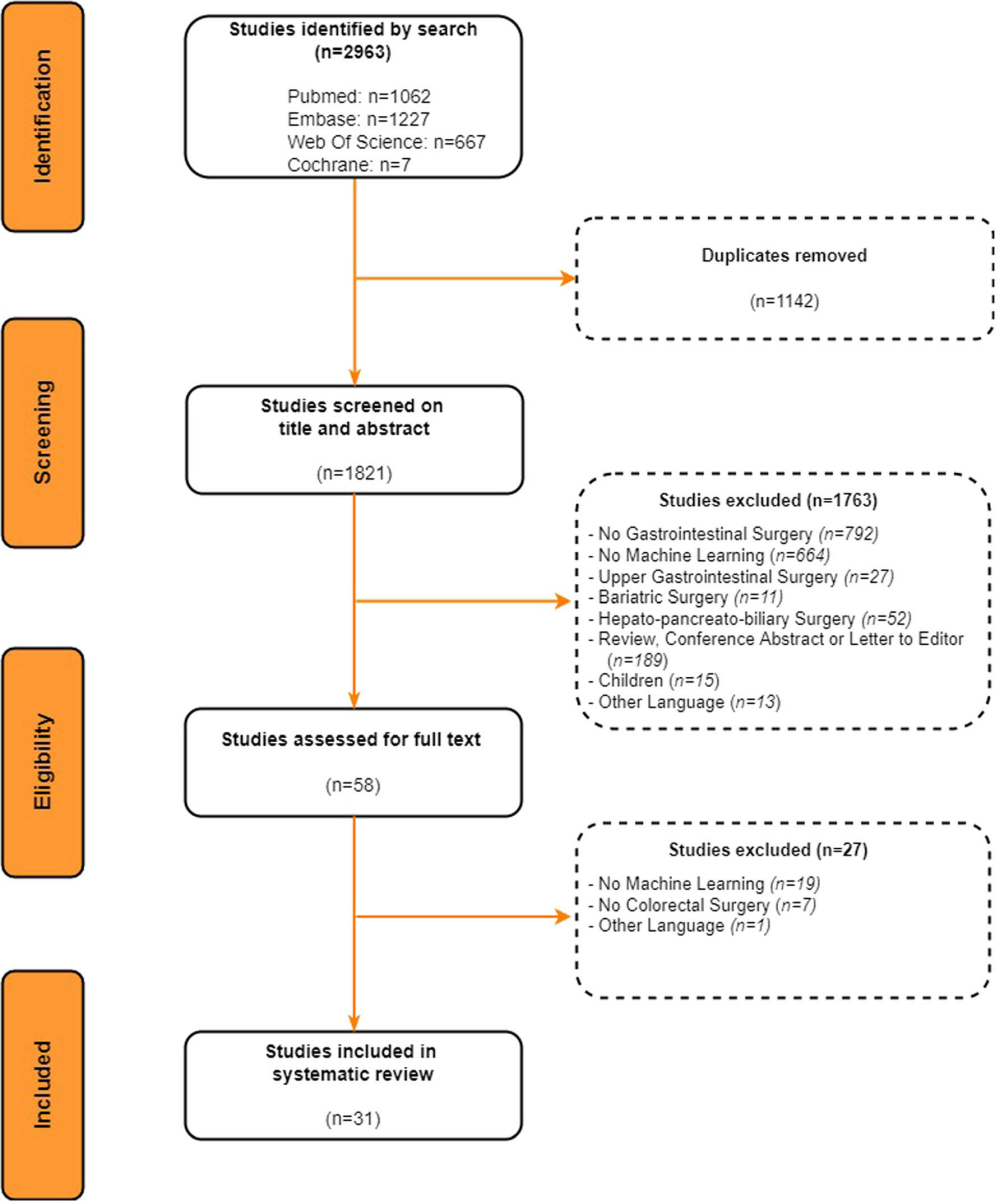
PRISMA flow chart of the study selection

The search strategy provided a total of 1821 studies after removal of duplicates (Fig. 1). Therefore, 1821 studies were screened for eligibility based on the title and abstract. After excluding 1763 studies, 58 studies remained for a full-text assessment. In the end, 31 studies were included in this systematic review.

### Machine learning models

Various machine learning algorithms have been applied to patients undergoing colorectal surgery. The frequencies of applied machine learning models were as follows: radiomics (*n* = 13), neural networks (*n* = 7), multiple machine learning (*n* = 6), random forest (*n* = 4), gradient boosting (*n* = 1).

### Surgical outcomes

Surgical outcomes of these machine learning models predominantly included prediction of the clinical staging and prognosis (*n* = 9), chemoradiotherapy response (*n* = 7), and postoperative complications (*n* = 7). Remaining studies involved prediction of diagnosis (*n* = 4), success of intervention (*n* = 2), and pre-and postoperative management (*n* = 2). An overview of key study characteristics is presented in Table 2.

**Table 2:** Key characteristics of included studies

Author	Year	Country	Patients	Age (mean)	Study design	Surgical procedures	Intervention (machine learning model)	Surgical outcomes	Internal validation method	External validation (yes/no)	Discrimination (ACC/AUC)	Calibration (yes/no)
Alvarez-Jimenez et al.	2020	USA	94	62	Retrospective Cohort	Total mesorectal excision	Radiomics	Prediction of tumour differentiation	Cross-validation	Yes	–/0.73	No
Lee et al.	2020	Korea	2019	62	Retrospective Cohort	Colectomy	CNN; RF	Prediction of metastasis	Cross-validation	No	–/0.70	No
Zhang et al.	2020	China	94	58	Retrospective Cohort	NS	Radiomics	Prediction of metastasis	Cross-validation	No	–/0.88	Yes
Eresen et al.	2020	USA	390	63	Retrospective Cohort	Colectomy	Radiomics; SVM	Prediction of metastasis	Cross-validation	No	–/0.83	No
Nakanishi et al.	2020	Japan	247	60	Retrospective Cohort	Total mesorectal excision	Radiomics	Prediction of metastasis	Bootstrapping	Yes	/0.91	Yes
Li et al.	2019	China	48	61	Retrospective Cohort	Colectomy	Radiomics; SVM	Prediction of metastasis	Cross-validation	No	0.86/0.87	No
Liang et al.	2019	China	108	55	Retrospective Cohort	Total mesorectal excision	Radiomics; SVM	Prediction of metastasis	Cross-validation	No	0.72/0.83	No
Li et al.	2020	China	207	62	Retrospective Cohort	Colectomy	Radiomics; SVM	Prediction of metastasis	Cross-validation	No	0.73/0.83	No
Dimitriou et al.	2018	UK	173	67	Retrospective Cohort	Colectomy	Multiple machine learning methods	Prediction of survival	Cross-validation	No	SVM–/0.95 RF–/0.92	No
Antunes et al.	2020	USA	104	63	Retrospective Cohort	Proctectomy	Radiomics; RF	Prediction of chemotherapy response	Cross-validation	No	0.71/0.71	No
Ferrari et al.	2019	Italy	55	65	Retrospective Cohort	Total mesorectal excision	Radiomics; RF	Prediction of chemotherapy response	Random split of dataset	No	–/0.86	No
Yuan et al.	2020	USA	91	56	Retrospective Cohort	Total mesorectal excision	Radiomics	Prediction of chemotherapy response	Random split of dataset	No	0.84/–	No
Boyne et al.	2020	Canada	1378	64	Retrospective Cohort	Colectomy	RF	Prediction of chemotherapy response	Bootstrapping	No	–/0.8	Yes
Fu et al.	2020	USA	43	54	Retrospective Cohort	Total mesorectal excision	Radiomics; CNN	Prediction of chemotherapy response	Cross-validation	No	–/0.73	No
Shaish et al.	2020	USA	132	63	Retrospective Cohort	Total mesorectal excision	Radiomics	Prediction of chemotherapy response	Cross-validation	No	–/0.80	No
Yi et al.	2019	China	134	52	Retrospective Cohort	Total mesorectal excision	Radiomics	Prediction of chemotherapy response	Random split of dataset	No	–/0.91	No
Weller et al.	2018	USA	4773	NS	Retrospective Cohort	NS	Multiple machine learning methods	Prediction of postoperative complications	Cross-validation	No	GBM–/0.64 RF–/0.6 SVM–/0.65	No
Chen et al.	2019	USA	13,399	58	Retrospective Cohort	NS	GBM	Prediction of postoperative complications	Cross-validation	No	–/0.82	No
Azimi et al.	2020	USA	208	58	Retrospective Cohort	Colectomy	Multiple machine learning methods	Prediction of postoperative complications	Bootstrapping	No	DT0.86/– RF0.93/– SVM0.81/– ANN 0.88/–	No
Bunn et al.	2020	USA	223,214	40	Retrospective Cohort	Appendectomy	Multiple machine learning methods	Prediction of postoperative complications	Random split of dataset	No	GBM–/0.93 RF–/0.96 SVM–/0.5	No
Adams et al.	2013	UK	76	61	Retrospective Cohort	NS	ANN	Prediction of postoperative complications	Random split of dataset	Yes	–/0.89	No
Wen et al.	2021	USA	5220	59	Retrospective Cohort	Anterior resection + Total mesorectal excision	RF	Prediction of postoperative complications	Cross-validation	Yes	–/0.85	No
Cao et al.	2020	Sweden	157	76	Retrospective Cohort	Emergency colectomy	RF	Prediction of mortality	Cross-validation	No	0.81/0.93	No
Akmese et al.	2020	Turkey	128	NS	Retrospective Cohort	Appendectomy	Multiple machine learning methods	Prediction of diagnosis	Random split of dataset	No	DT0.81/– GBM0.95/– RF 0.93/– SVM 0.80/– ANN 0.65/–	No
Hsieh et al.	2010	Taiwan	180	40	Retrospective Cohort	NS	Multiple machine learning methods	Prediction of diagnosis	Cross-validation	No	RF 0.96/0.98 SVM 0.93/0.96 ANN 0.91/0.91	No
Yoldas et al.	2011	Turkey	156	30	Retrospective Cohort	NS	ANN	Prediction of diagnosis	Random split of dataset	No	–/0.95	No
Prabhudesai et al.	2007	UK	60	25	Retrospective Cohort	NS	ANN	Prediction of diagnosis	Random split of dataset	No	0.98/–	No
Gardiner et al.	2004	UK	72	NS	Retrospective Cohort	Anterior sphincter repair	ANN	Prediction of intervention success	Random split of dataset	No	0.93/–	No
Manilich et al.	2012	USA	3754	38	Retrospective Cohort	proctocolectomy	RF	Prediction of intervention success	Random split of dataset	No	NS	No
Curtis et al.	2019	UK	668	70	Retrospective Cohort	NS	ANN	Prediction of pre- and postoperative management	Cross-validation	No	–/0.86	No
Francis et al.	2015	UK	275	NS	Retrospective Cohort	NS	ANN	Prediction of pre- and postoperative management	Random split of dataset	No	–/0.75	No

*ACC* accuracy, *AUC* area under the curve, *NA* not applicable, *NS* not specified

### Methodological quality assessment

**Fig. 2:**
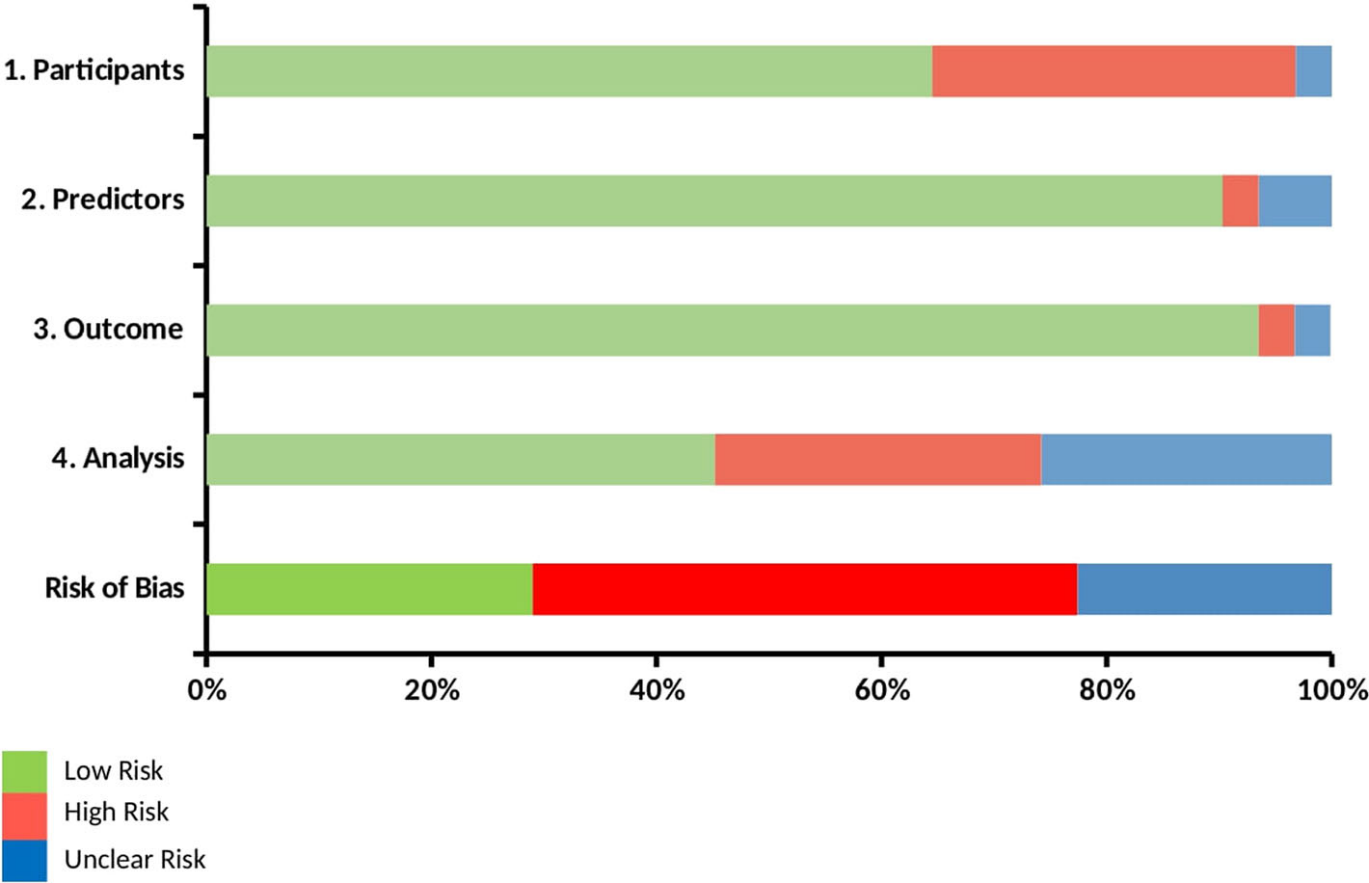
Methodological assessment of ML models, according to the Probast risk of bias tool

Based on the Probast tool, the majority of studies received a low risk of bias score for the predictors and outcome domains. For most studies, the participants and analysis domains have received unclear or high risk of bias scores due to inappropriate inclusion criteria or measures to account for overfitting and missing data. Therefore, a low overall bias was given for 29% of the studies, whereas 48% of the studies received an unclear overall bias. Additionally, a high overall bias was decided for 23% of the studies (Fig. 2).

### Model validation

For internal validation of machine learning models, most studies used cross-validation (*n* = 17), a random split of the dataset (*n* = 11), or bootstrapping (*n* = 3). External validation was performed in four studies (13%), including two radiomics, one ANN, and one random forest model. The discriminative ability (AUCs) of these models ranged between 0.64 and 0.9, and the calibration was reported for one machine learning model.

### Predictive performance

The AUCs of machine learning models for predicting clinical staging and prognosis have ranged between 0.7 and 0.95, whereas the accuracies were discovered to be between 72 and 86% [21–29]. For the prediction of chemotherapy response, AUCs between 0.71 and 0.91 were discovered, accuracies varied between 71 and 84% [30–36]. In the machine learning group for prediction of postoperative complications, AUCs have ranged from 0.6 and 0.96, additionally, accuracies were found to be between 81 and 93% [37–43]. For machine learning models predicting appendicitis cases, AUCs varied from 0.91 to 0.95, and accuracies ranged between 65 and 98% [44–47]. In the group for prediction of intervention success, AUCs were up to 0.93 [48, 49]. For prediction of pre-and postoperative management, machine learning models showed AUCs up to 0.86 [50, 51]. Calibration was described for three models (10%), in which two studies used a Hosmer–Lemeshow test, and one study used a calibration plot only. Additionally, one study did not use AUC or accuracies to describe predictive performance of the machine learning model.

## Discussion

This review illustrates the capabilities of machine learning in predicting several surgical outcomes for patients undergoing colorectal surgery. In this study, promising discriminative abilities of applied ML models have been discovered, especially for radiomic models.

Nine studies have used machine learning algorithms to predict the course of disease with accuracies ranging between 70 and 90%. Radiomics models have shown highest accuracies in these predictions. Theoretically, the use of ML could improve pre-operative decision-making for patients undergoing colorectal surgery, eventually enabling individualized surveillance for patients. For patients with high risks of metastasis, treatment decision such as minimal or aggressive surgery could be reconsidered for optimal surgical outcomes. However, most studies included small cohorts, this might give rise to the problem of overfitting, in which the ML model is overly adjusted to the training dataset and is unable to perform well on the test set [52, 53]. Although measures such as cross-validation and feature selection might help, this problem could be solved by including an external validation cohort [54].

Seven studies have applied machine learning to predict response to neoadjuvant chemoradiation therapy (nCRT) with accuracies between 71 and 91%. Radiomics appeared to perform this prediction with the highest accuracies. Although chemoradiotherapy has already shown improved outcomes for patients with advanced rectal cancer, incomplete therapy response and overtreatment of nCRT could occur [55]. Surgeons experience difficulties in determining patients who would completely respond to nCRT [56]. By using machine learning, surgeons could improve risk stratification, and decide to tailor therapy to patients with predicted nCRT response. This might eventually enable personalized decision-making for every patient, preventing unnecessary hospital stays and costs.

Seven studies have attempted to predict postoperative complications. Accuracies of ML models have ranged from 47 and 96%, in which random forests had the best predictive performance. Ideally, colorectal surgeons could use machine learning models to accurately predict postoperative complications for every patient. Subsequently, early discharge, enhanced monitoring or prophylactic steps could be implemented based on the predicted risk of complications. In addition, one study developed a predictive model for mortality in patients undergoing acute abdominal surgery [43]. This could potentially be helpful for clinical decision-making in acute surgery. Nonetheless, these ML studies have primarily included preoperative risk factors for postoperative complications. Previous studies have already indicated that postoperative complications are dependent upon several preoperative, intraoperative, and postoperative risk factors [57]. Therefore, more datasets are required to reveal essential intraoperative and postoperative factors for the prediction of postoperative complications.

For predicting patients with acute appendicitis, ML models have performed with accuracies up to 98%. Akmese et al. have demonstrated that ML could be applied with web-based interfaces, with internet as the only necessary criteria. Prabhudesai et al. have discovered that neural networks are able to predict appendicitis cases better than clinicians. These two findings may suggest that ML models could be practical and accurate tools for improving surgical decision-making. With proper use, surgeons could diagnose faster and prevent unnecessary appendectomies.

Although high accuracies have been found for machine learning models within this review, it seems that some uncertainties are still present. External validation was missing in most of the studies (87%), indicating that most machine learning models have not been applied to data from external hospital settings. However, external validation is crucial to demonstrate the generalizability of machine learning models [58]. Additionally, calibration was not reported in most studies (90%), while calibration reflects the similarity between predicted risks and the true observed risks [59]. Poor calibration indicates that the machine learning model is under- or overestimating the desired outcome.

This review has some limitations. Due to the heterogeneity in methodologies of studies, a comparative meta-analysis of ML models was not possible. Additionally, a number of studies have not described predictive performances of ML models in ACC or AUCs, possibly leading to an over- or underrepresentation of actual discriminative abilities.

Future studies should focus on the external validation of ML models. Since external validation is important for the generalizability of machine learning algorithms, gaining this validation could facilitate the introduction of machine learning in daily clinical practice. However, large-scale datasets are required for this external validation, existing patient databases could be used to fulfill this need [60]. With proper use of these data, surgeons may achieve personalized decision-making for patients undergoing colorectal surgery. In addition, the calibration of machine learning models should be demonstrated in future studies to represent the extent of consensus between predicted outcomes and outcomes in the clinics.

In conclusion, this review shows the promising potential of ML in predicting various surgical outcomes for patients undergoing colorectal surgery. However, clinical implementation is required to demonstrate the contribution of ML within daily practice. The use of large patient databases may be required to fulfill the need for calibration and external validation.

## Supplementary Information

Below is the link to the electronic supplementary material.

Supplementary file1 (DOCX 28 kb)
